# Laparoscopic *vs* Open Ultrasound of the Liver:  An *in vitro* Study

**DOI:** 10.1155/1996/71637

**Published:** 1996

**Authors:** P. J. Cozzi, J. L. McCall, J. O. Jorgensen, D. L. Morris

**Affiliations:** UNSW Department of Surgery The St.George Hospital Kogarah Sydney NSW 2217 Australia

## Abstract

Intra-operative contact ultrasound is a sensitive method of detecting liver tumours. The aim of this study was to compare the sensitivity of open contact ultrasound (OUS) of the liver with laparoscopic contact ultrasound (LUS). Hypoechoic “lesions” were created in 5 fresh pig livers by inserting 28 grapes via small incisions in the inferior surface. The size (range 8–25 mm) and location of each grape was recorded. Scanning was undertaken in random order by two experienced independent observers with no knowledge of the size, number or position of the lesions, using an Aloka 650 series scanner and 7.5 MHz probes. The crude sensitivity with OUS was 96% and 100% respectively for the two observers, and 92% for each with LUS. One grape was interpreted as 2 seperate grapes on LUS by one observer. Absolute sensitivity (grapes identified in the correct location) was 86% and 93% respectively with OUS and 79% for each observer with LUS.

LUS was almost as sensitive as OUS in this model of hepatic metastases.


*HPB Surgery*, 1996, Vol.10, pp. 87–89
Reprints available directly from the publisher
Photocopying permitted by license only

© 1996 OPA (Overseas Publishers Association)
Amsterdam B.V. Published in The Netherlands
by Harwood Academic Publishers
Printed in Malaysia

### Laparoscopic *vs* Open Ultrasound of the Liver: An *in vitro* Study

P. J. COZZI, J. L. McCALL, J.O. JORGENSEN and D. L. MORRIS

UNSW Department of Surgery, The St.George HospitalKogarah Sydney NSW 2217 Australia

*(Received 25 June 1995)*

Intra-operative contact ultrasound is a sensitive method of detecting liver tumours. The aim of this study was to compare the sensitivity of open contact ultrasound (OUS) of the liver with laparoscopic contact ultrasound (LUS). Hypoechoic "lesions" were created in 5 fresh pig livers by inserting 28 grapes via small incisions in the inferior surface. The size (range 8–25 mm) and location of each grape was recorded. Scanning was undertaken in random order by two experienced independent observers with no knowledge of the size, number or position of the lesions, using an Aloka 650 series scanner and 7.5 MHz probes. The crude sensitivity with OUS was 96% and 100% respectively for the two observers, and 92% for each with LUS. One grape was interpreted as 2 seperate grapes on LUS by one observer. Absolute sensitivity (grapes identified in the correct location) was 86% and 93% respectively with OUS and 79% for each observer with LUS.
LUS was almost as sensitive as OUS in this model of hepatic metastases.

KEY WORDS: Laparoscopy intra-operative ultrasound liver metastases

### INTRODUCTION

The benefits of hepatic resection for treatment of primary and secondary malignancy are well documented [1,2]. Selecting patients for resection requires accurate pre-operative and intra-operative evaluation. Of all the modalities currently available to evaluate the liver, intra-operative ultrasound (IOUS) is the most accurate[3]. High resolution images are obtained using 5 and 7.5 MHz contact probes, capable of detecting lesions of 5 mm or less in diameter[4].

Until recently IOUS of the liver was undertaken at laparotomy but with the advances in laparoscopic surgery and the development of laparoscopic ultrasound probes it is now possible to perform laparoscopic ultrasonography (LUS). LUS has recently been advocated as a means of staging patients with intra-abdominal malignancy and avoiding unnecessary laparotomy in patients with advanced disease[5,6]. However, the accuracy of LUS has not been validated against open IOUS (OUS) for the detection of metastatic liver disease. In this study LUS and OUS have been compared using a bench top model of hepatic metastases.

### MATERIALS AND METHODS

### *Hepatic Metastases Model*

Five fresh pig livers were used. Each liver was marked out into eight, approximately equal, segments using diathermy on the superior surface. "Lesions" were created by implantiong grapes (28 in all), ranging in diameter from 0.8 to 2.5 cm, via separate small incisions in the inferior surface of the livers. The size and location of each grape was recorded. The entry tracts were filled with an iso-echoic solution (K-Y Jelly. Johnson and Johnson.UK) to exclude gas, then oversewn. The resulting lesions were hypo-echoic with

*Correspondence to:* Professor D.L. Morris, UNSW Department of Surgery, The St. George Gray Street Kogarah Sydney NSW 2217 Australia Phone: (61 2) 350 2070 Fax: (61 2) 350 3997

a hyper-echoic rim, similar in appearance to some hepatic metastases from colorectal carcinoma.

### *Scanning*

An Aloka series 650 scanner (Aloka Co. Ltd. Japan) was used with the 7.5 MHz linear array probe for OUS and the 7.5 MHz Electronic Linear Probe (UST-5521-L-7.5) for LUS. Scanning was undertaken independently by two surgeons. Both were experienced in OUS and in laparoscopy but neither had previous experience with LUS. Both surgeons were blinded as to the number, size and position of the lesions.

LUS was undertaken first. For this, the livers were placed individually, superior up, in a light proof zero laparoscopic training box (Lap trainer, Big City Productions, Sydney). A 10 mm 0 degree video-laparoscope was used and the LUS probe introduced via a second 10 mm port. A third 5 mm port was used to introduce a grasper to manipulate the liver. Each liver was scanned and the size and location of detected lesions was recorded.

The livers were then re-scanned, in random order, using OUS on the benchtop. During this, the observers were permitted to use palpation, thus simulating operative conditions. The size and location of detected lesions was again recorded.

### *Statistical Analysis*

The crude sensitivity (ability to detect lesions) and the absolute sensitivity (ability to detect and correctly place lesions) of the two techniques was determined. Accuracy of size measurement with each technique was calculated by the method of Bland and Altman [7].

### RESULTS

The two observers detected 27 and 28 of the 28 lesions by OUS, giving a crude sensitivity of 96% and 100% respectively (Fig.1). Both observers detected 26 of the

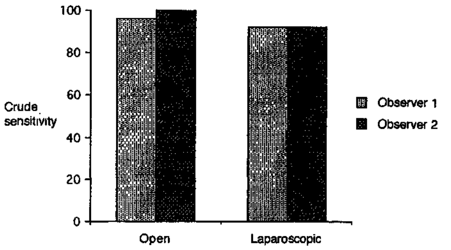

**Figure 1** Sensitivity of operative (contact) ultrasound to detect experimental liver lesion.

28 lesions by LUS with a crude sensitivity of 92% (Fig.1). One observer incorrectly identified a single lesion as 2 separate lesions on LUS. Absolute sensitivity (lesions identified and placed in the correct liver segment) was 86% and 93% respectively for the two observers using OUS, and 79% for both observers using LUS (Fig. 2). Absolute sensitivity was not

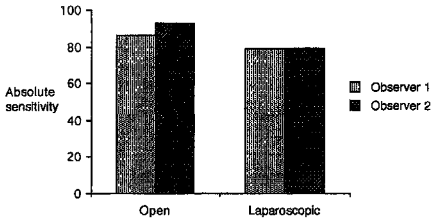

**Figure 2** Sensitivity of laparoscopic ultrasound to detect experimental liver lesions.

different for lesions of various size with either technique. Lesions detected with OUS were within –6.5 and +3.5 mm of actual size, and those detected with LUS were within –6.3 annd +5.3mm of actual size, at the 95% confidence limits[7].

### DISCUSSION

This study has demostrated that LUS is almost as good as OUS in the detection of liver lesions in an *in vitro* model. The sensitivity of each method is well within the expected range for IOUS from previous studies, both *in vivo*[3,5,6] and *in vitro*[4].

Several factors may have been responsible for the small difference between OUS and LUS in this study. Both observers were experienced in OUS of the liver but not LUS. Any resulting bias would tend to favour the results of OUS. Perhaps more importantly, OUS permits simultaneous palpation which aids in the diagnosis of small superficial or subcapsular lesions. Furthermore, whilst the quality of image is comparable, obtaining complete coverage of all segments of the liver may be more difficult with the laparoscopic probe fulcrummed against the abdominal wall. These difficulties can be overcome to a certain extent by taking care to maintain contact along the length of the probe, scanning from below as well as above and changing angles by swapping the probe to other ports.

Some important difference exist between the model used in the present study and the situation *in vivo* In the *ex-vivo* liver there are no vascular landmarks such as hepatic veins and Glissonian sheaths to define anatomical segments, hence the need for surface markings on the liver capsule. The exsanguinated liver is also somewhat more dense and echogenic than the perfused liver and this has the effect of reducing image quality. Conversely, grapes produce an easily recognisable lesions with a hyper-echoic rim and iso or hypo-echoic centre. Hepatic metastases may produce hyper-hypo-or iso-echoic images and are not always easy to detect.

Although assessing lesion size is less important than detecting their presence and location, it is an indirect measure of the accuracy and clarity of the ultrasound image. In this regard both OUS and LUS were equally satisfactory with most lesions assessed as being within 5 mm of their actual size.

In a recent series additional information obtainaed solely from LUS led to a change in surgical approach in 5 of 19 patients with liver lesions[5]. Miles *et al.*[6] detected unexpected disease in 6 of 7 patients with hepatic malignancy. In Bismuth's series of 77 patients with primary liver tumours submitted to surgery, 7 patients were found to have inoperable lesions based on findings with IOUS[3]. All these patients had under gone pre-operative ultrasonography, computerised tomography (CT) and selective hepatic angiography with delayed portal venous phase radiology (angio CT) prior to surgery. Other authors have suggested that ultrasound, CT and magnetic resonance imaging have a sensitivity of less than 80% for the detection of number and extent of liver metastases from colorectal carcinoma[8,9]. Angio CT has added much to the pre-operative staging of these patients but in addition to the 10% false negative rate described by Bismuth[3], false positives may occur in as many as 15% of patients[10].

Diagnostic laparoscopy, combined with LUS will prevent some patients with unresectable disease from undergoing unnecessary laparotomy. LUS also enables equivocal findings on angio CT to be evaluated further without laparotomy.

This study demonstrates that LUS is almost as good as OUS for the detection of liver lesions, confirming its usefulness in staging patients with hepatic malignancy.

### REFERENCES

1. Adson M.A., Heerden J.A., (1980) Major hepatic resections for metastatic colorectal cancer. *Ann Surg.*, 576–583.
2. Greenway B. (1988) Hepatic metastases from colorectal cancer; resection or not. *Br. J. Surg.*,**75**, 513–519.
3. Bismuth H., Casting D. and James Garden O. (1987) The Use of Operative Ultrasound in Surgery of Primary Liver Tumors. *World Journal of Surgery.*, **11**, 610–614.
4. Thomas W.M., Morris D.L. and Hardcastle J.D. (1987) Contact ultrasonography in the detection of liver metastases from colorectal cancer: an in vitro study. *Br.J.Surg.*, **74**, 955–956.
5. Cuesta M.A., Meijer S., Borgstein P.J., Sibinga Mulder L. and Sikkenk A.C. (1993) Laparoscopic ultrasonography for hepatobiliary and pancreatic malignancy.*Br.J.Surg.*, **80**, 1571–1574.
6. Miles W.F.A. Paterson-Brown S. and Garden O.J. (1992) Laparoscopic contact hepatic ultrasonography. *Br.J.Surg.*, 419–420.
7. Bland M.J., Altman D.G. (1986) Statistical methods for assessing agreement between two methods of clinical measurement. *The Lancet.*, **8**, 307–310.
8. Goulet R.J., Seekri I. and Inman M. *et al.* (1990) The diagnosis and definition of hepatic malignancies by use of arterially enhanced computerized tomographic scanning. *Surgery.*, **108**, 694–701.
9. Boutkan H., Meijer S. and Cusesta M.A. *et al.* (1991) Intraoperative ultrasonography of the liver: a prerequisite for surgery of colorectal cancer? *Neth. J. Surg.*, **43**, 89–91.
10. Soyer P., Lacheheb D., Levesque M. (1993) False positive CT portography; correlation with pathological findings. *Am. J. Roenterol.*, **160**, 285–289.

